# Root Community Traits: Scaling-Up and Incorporating Roots Into Ecosystem Functional Analyses

**DOI:** 10.3389/fpls.2021.690235

**Published:** 2021-07-22

**Authors:** Ruili Wang, Guirui Yu, Nianpeng He

**Affiliations:** ^1^College of Forestry, Northwest A&F University, Yangling, China; ^2^Key Laboratory of Ecosystem Network Observation and Modeling, Institute of Geographic Sciences and Natural Resources Research, Chinese Academy of Sciences, Beijing, China; ^3^College of Resources and Environment, University of Chinese Academy of Sciences, Beijing, China; ^4^Institute of Grassland Science, Northeast Normal University and Key Laboratory of Vegetation Ecology, Ministry of Education, Changchun, China

**Keywords:** root trait, community-level trait, species-level trait, ecosystem functioning, scale-matching

## Introduction

Plant roots are important organs responsible for the physical support and for the acquisition of nutrients and water from the soil, which is necessary for plant growth and reproduction. Thus, the morphological, structural, physiological, and biochemical traits of roots theoretically play crucial roles in driving a series of physiological and ecological functions on different levels, from individuals to ecosystems (Bardgett et al., 2014; Laliberté, 2017; McCormack et al., 2017; Freschet et al., 2021). The latest study of Freschet et al. (2021) stated that root traits were central to the maintenance of multiple ecosystem processes and functioning, especially the transformation and circulation of elements and mineral/organic compounds across the spheres. These previous studies provide us the theoretical basis for the linkages between root traits and the different aspects of ecosystem functioning, such as gross primary productivity (GPP) and nutrient-use efficiency (NUE).

## Substantial Progress in the Study of Root Trait Variation

Root traits are initially studied in agricultural systems for developing crops with superior growth and high productivity. In recent decades, root traits of natural communities have attracted increasing interest, particularly in the intra-and inter-species variation, trait covariation, strategies of nutrient acquisition, and mechanisms of species coexistence (Eissenstat, 1992; Lambers et al., 2008; Ma et al., 2018; Kong et al., 2019; Freschet et al., 2021). The scientific community has gradually recognized that compared with coarse roots, fine (<2 mm diameter) or absorptive roots (first- to third-order fine roots) are more active in the acquisition of resources at the species level (Pregitzer et al., 2002; McCormack et al., 2015). Classifying roots into functional modules is a key step that promotes the progress in ecological research largely based on root traits, leading to the identification of general principles of root variation among various plant species, environments, and root orders (McCormack et al., 2015; Iversen et al., 2017).

Comparative root ecology has recently shown that plant species are able to regulate the plasticity of their root traits to adapt to the external environment by modulating the morphology, architecture, and microbial associations of fine roots via trade-off between nutrient acquisition efficiency and investment (Kramer-Walter et al., 2016; Ma et al., 2018). For example, in woody plants, the diameter of first-order roots decreases from plants in tropical (stable habitats) to desert (infertile and highly seasonal habitats) biomes, which is accompanied by a reduced reliance on mycorrhizae and thinner roots allowing for an increase in carbon-use efficiency that can aid the colonization of new habitats (Ma et al., 2018). The development of global databases of root traits across different species, biomes, and environmental gradients (such as the Fine-Root Ecology Database, http://roots.ornl.gov) using standardized protocols may, thus, be vital for improving our predictive capacity across different ecological scales in the future.

## Discussion

### How to Link Root Traits With Ecosystem Functioning?

Despite the aforementioned substantial advances toward a greater understanding of intra- and inter-species root trait variation, the importance of root traits in ecosystem-level functioning, particularly carbon and nutrient cycling, is increasingly recognized but still not well-understood (Freschet et al., 2021). Understanding and predicting the effects of plant functional traits on certain key ecological processes has been coined as the “holy grail” in ecological research (Lavorel and Garnier, 2002). However, root traits remain underrepresented or non-parameterized in studies on ecosystem models and terrestrial biosphere functioning (Warren et al., 2015), partly because the mismatch between traits and functions is apparent between species and natural communities. Little evidence of the links between root functional traits and community or ecosystem-level functioning has been reported or verified in complex natural communities (van der Plas et al., 2020; Freschet et al., 2021); therefore, scaling up the links between them from organ or species to ecosystem levels may lead to large uncertainties.

**Figure 1:**
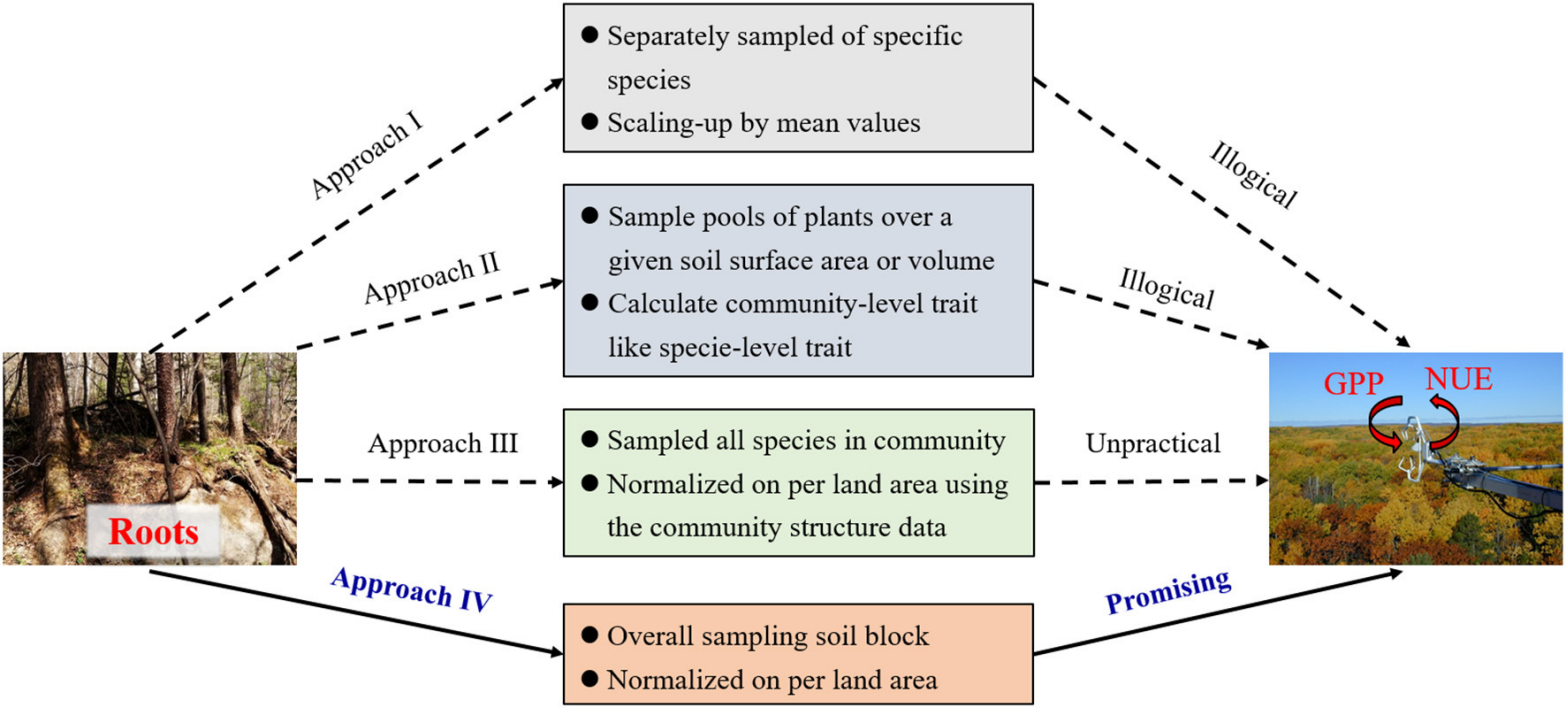
Illustration of the four approaches used for linking root traits and ecosystem functioning in forests. GPP, gross primary productivity; NUE, nutrient-use efficiency. Approach I: root trait of dominant species or community-weighted means; Approach II: pooled-species trait; Approach III, ecosystem traits raised by He et al. (2019); Approach IV: root community traits (per unit land area). The advantages of Approach I and II are simple to conduct on the local scale and root trait data of each species is relatively easy to collect worldwide. However, mismatched units or dimensions lead to illogical linkages between root traits and ecosystem functioning in these two approaches. Although the Approach III could fill the gap of scale-matching between root traits and ecosystem functioning, it requires systematic measurements of all species within a community. Furthermore, some basic conversion parameters, such as root biomass and area per unit land area, are difficult to obtain. Through overall sampling soil block, Approach IV directly obtains the community-level root traits per unit land area, which not only has the potential to bridge the mechanistic linkages between root traits and ecosystem functioning, but also is feasible at the large scale.

Identifying the links between root traits measured at the organ level and the ecosystem-level functioning, and even integrating these root traits into ecological models and remote sensing techniques are great challenges in ecological studies (Laliberté, 2017; McCormack et al., 2017; Freschet et al., 2021). One common method used in previous studies is to calculate the trait values of the dominant species by averaging or weighting all species' abundances in a community (e.g., community-weighted mean, CWM) to represent the values for an entire plant community (Approach I, Figure 1). Following this, the variation in specific root traits and its consequences on higher-level processes and functions could be identified (Lavorel and Garnier, 2002; Violle et al., 2007). This method is feasible for assessing community-level root traits worldwide with the help of the databases of each species' root traits and community structure. However, in practice, three vital difficulties must be overcome at large scales when trying to link community-level root traits with ecosystem functioning. First, it is challenging and laborious to obtain the values of root abundance (or biomass) for each species in natural communities, which is a basic conversion factor in CWM methods. Although plant dominance is typically assessed according to aboveground features (e.g., Mokany et al., 2008; De Long et al., 2019), belowground organs of individual species may not scale proportionally in relation to their aboveground dimension. This case is especially important in ecosystems where most biomass is allocated belowground, e.g., grasslands and shrubby biomes (Ottaviani et al., 2020). Secondly, these traditional scaling-up approaches are based on the assumption of linear or approximately equal species contributions (Reichstein et al., 2014), and few studies have directly tested and quantified the linkages between organ-level root traits and ecosystem functioning, especially in the complex natural ecosystems (van der Plas et al., 2020; Freschet et al., 2021). As a result, major research challenges still face ecologists when studying the interface between root traits and ecosystem functioning (Freschet et al., 2021). Thirdly and most importantly, mismatched units between ecosystem functioning and CWMs decouple their relationships. Functions at the ecosystem level are generally estimated based on land area by using eddy-flux observations, remote sensing, or ecological modeling. By contrast, the units of root traits based on CWMs remain the same as those of measured plant organ traits, such as root element content (g kg^−1^) and specific root length (SRL, mm^2^ mg^−1^). Another method proposed to estimate the community traits is pooled-species approach (Approach II, Figure 1), which pools of plants are sampled over a given soil surface area or soil volume and the community-level functional parameters (e.g., SRL; root tissue density, RTD) are directly measured (Klumpp and Soussana, 2009; Prieto et al., 2016). Compared with the CWM approach, the pooled-species approach is far less time consuming without need to estimate root abundance of each species. However, the pooled-species approach also fails to solve the problem of mismatched units of root traits with ecosystem properties. Such shortcomings of mismatched units or scales limit the development of these traditional methods and their application in ecological models (Warren et al., 2015; McCormack et al., 2017).

Developing a new methodology for quantifying community-level root traits per unit of land area is both theoretically and practically essential to better incorporate root traits into ecological models (He et al., 2019; Liu et al., 2021). This new concept of ecosystem traits (He et al., 2019) should be helpful, because the traits of ecosystems or plant communities are defined as the traits or quantitative characteristics of organisms at the community level and expressed as the intensity (or density) normalized per unit land area (Approach III, Figure 1). Foliar area or biomass per unit land area of specific species has been used to calculate foliar physiological parameters of ecosystem traits, such as total stomatal number per unit ground surface area (number m^−2^), as well as to identify the tight links with the productivity and water-use efficiency of an ecosystem (Wang et al., 2015). The concept of ecosystem traits inspires us to rethink the sampling methods to resolve these questions for natural ecosystems.

However, Approach III will result in larger challenges in calculating community-level root traits because obtaining the basic conversion parameters for scaling up, such as root biomass or area of each plant species, is more difficult for roots than for leaves in natural communities. All evidences support the use of holistic sampling methods, i.e., using root cores or soil blocks under a given soil surface area or volume to identify the morphological, chemical, and physiological traits of roots, and then normalizing the data per unit land area (i.e., root community traits, Approach IV, Figure 1). The advantage of holistic sampling is that we can directly obtain the traits of community-level root entities, which enables us to match the units with ecosystem functioning and to integrate data from field traits with the approaches and technologies used in macroecology. Many studies have used root cores to determine root traits, but most of them only investigated root biomass per unit land area or calculated functional parameters like species-level traits (e.g., Klumpp and Soussana, 2009; Prieto et al., 2016). The concept of root community traits urges us to measure and identify the total spatial and temporal variation in root traits, which may enable us to better link the traits with ecosystem functioning (e.g., GPP or NUE) in the natural ecosystems at a large scale.

### What Is Promising for Root Community Traits in Ecosystem Studies?

Here, we define root community traits as the overall morphological, chemical, and physiological traits of roots in a natural community, and they are normalized per unit land area. The new concept of root community traits is important for plant growth and production for each specific trait or all of them jointly in natural communities. For example, at the species level, SRL, i.e., the length of roots per unit biomass, can indicate the economics of root investment, and high SRL often indicates a high efficiency of nutrient uptake by roots and a high respiration rate (Reich, 2014). As a community-level root trait, the total root length per unit land area (i.e., root length density, RLD) could be quantified to indicate the nutrient uptake capacity of a community by belowground roots, and it could account for the variation in ecosystem processes, particularly belowground cycling of carbon and nutrients. Similarly to RLD, we also could estimate the density of total root biomass, area, and chemical contents per unit land area. Therefore, the parameters of root community traits should be widely used in studies at the ecosystem level. We suggest the following four practices in future studies: (1) determining the relationships between community-level root traits and the environment at a large scale and investigating empirical relationships between root traits and ecosystem-level functioning are required to promote ecological models of vegetation dynamics; (2) studies of traditional root traits should be integrated with macro-ecological studies using new technologies (e.g., ground penetrating radar and remote sensing); (3) new perspectives should be offered for understanding the relationships between above- and belowground traits. Whether above- and belowground traits co-vary or exhibit coordinated responses to a changing environment has been intensively debated and remains controversial (Weemstra et al., 2016; Wang et al., 2017). Within a community, tree canopies tend to extend fully and capture more light for photosynthesis to meet the demand for the growth of stems and roots, and in turn, belowground roots provide anchorage and resource acquisition. The concept of root community traits enables us to re-examine the relationship between roots and shoots from the perspective of above- and belowground plant communities; (4) with the help of molecular and DNA sequencing-based techniques, we can determine the belowground dominance of individual species on the basis of land area. Thus, the mechanisms of community structure assembly and productivity optimization in nature could be explored from a new perspective.

In conclusion, this new idea of root community traits may help us re-examine the multiple roles of plant roots in community assemblages and ecosystem processes, and it can help us resolve the difficulties of traditional scaling-up approaches. In the combination with some new observation technologies (e.g., ground penetrating radar, multispectral and X-ray images), we may track the dynamics of entire root systems over different spatial and temporal scales. More importantly, this new concept of root community traits can help us incorporate root traits into the investigations of ecosystem functioning at large scales, as well as to improve ecological models, particularly for productivity, nutrient acquisition, and soil carbon cycling.

## Author Contributions

NH and GY designed the research. RW, GY, and NH wrote the manuscript. All authors contributed to the article and approved the submitted version.

## Conflict of Interest

The authors declare that the research was conducted in the absence of any commercial or financial relationships that could be construed as a potential conflict of interest.
